# Editorial

**Published:** 1898-06

**Authors:** 


### Editorial.

### MEETING OF THE ALUMNI ASSOCIATION OF THE SOUTHERN MEDICAL COLLEGE.

As previously announced in THE SOUTHERN MEDICAL RECORD, the meeting of the Alumni Association of the Southern Medical College will take place July 20, 21 and 22.

The alumni of the college number about five hundred, while the number in Atlanta alone is beyond fifty. The institution should celebrate the close of a score of years since the original charter was granted. The love of every student for his *alma mater* is strengthened and sustained by the past record of the college. At no time in its history did the Southern Medical College stand for anything but clean, unhampered methods. Since its organization the Southern Medical College has grown and developed until now the record which it has made should be an incentive to the graduates to perpetuate its name in the new century as the Alumni Association of the Southern Medical College.

We are sure that no graduate of the college will fail to attend this meeting, if practicable. Notice by letter has been given those whose addresses were known, but necessarily many have been omitted. The exchanges are asked to copy this and to help the dissemination of the news in this way.

A medical society of alumni is the purpose and scope of the present reunion. The many practitioners who fail to report cases to the medical societies now in existence have led to the impression that an informal interchange of experiences may bring out their latent talent. The Kimball House is prepared to furnish a convenient room for the meeting and will be the headquarters. Many alumni will be in the city on account of the Confederate Veterans' reunion.

The papers which will be presented are short reports of the cases and suggestive remarks thereupon. The discussion is especially desired.

The present occasion is an auspicious time. The alumni of the Southern Medical College are requested to co-operate, one and all, to make this meeting a success. The alumni of many of the medical colleges are organizing for mutual improvement, and why should not the Alumni Association of the

Southern Medical College continue their present body, and in so doing improve the ethics and the standard of the profession?

The Southern Medical Society was the name of the medical society of the undergraduates of the Southern Medical College. The president for next year has doubtless been elected, and we would suggest to him and its members the continuation of this society also.

---

If it will be of any value to the profession in enabling the busy practitioner to keep a record of cases, we would mention an especially useful chart. Write to the Imperial Granum Food Company, New Haven, Conn., for sample copies of their new "Nursing World Fever Chart," for recording the vital signs and other information relating to the baths given in the treatment of fever cases. It is very complete and will be found especially useful in typhoid fever. We like it because in recording temperature no zigzag lines are needed.

---

ERRATUM.—On page 280 of the May issue of THE RECORD, in middle of page, read one-third fare, as follows:

"A meeting of the Alumni Association of the Southern Medical College is called for the 20th, 21st and 22d of July, 1898. For further information write to the undersigned or to the secretary, Dr. J. R. Shannon, Cabaniss, Ga. Railroad rates *one-third* fare on all roads, due to the meeting of the Confederate Veterans' reunion.

"J. McF. GASTON, JR., A.M., M.D., President."

---

### THE MEDICAL COLLEGES UNITED.

It is said that the unexpected always happens. It is very certain that nothing could have been more unexpected than the recent coalition of the Atlanta and the Southern Medical Colleges to form the Atlanta College of Physicians and Surgeons. The spirit of rivalry always existing between the two institutions had grown more and more marked, and was very near to causing a schism in the body medical of this city. The ease and facility with which old wrongs were adjusted, old grievances wiped out, all differences settled and consolidation effected, clearly demonstrated the superficiality of prejudice on both sides, especially when measured against the general good of the profession.

The combination is necessarily a strong one, and it requires no gift of prophesy to foretell that the Atlanta College of Physicians and Surgeons is destined to become one of the leading institutions of its kind in the South, and one of the thriftiest handmaids in the the service of science.

The new faculty is as follows:

A. W. Calhoun, M.D., President, and Professor of Diseases of the Eye and Ear, Nose and Throat.

W. S. Kendrick, M.D., Dean, and Professor of the Principles and Practice of Medicine.

Wm. Perrin Nicolson, M.D., Professor of Anatomy and Clinical Sugery.

H. P. Cooper, M.D., Professor of Anatomy and Clinical Surgery.

W. F. Westmoreland, M.D., Professor of Principles and Practice of Surgery.

J. S. Todd, M.D., Professor of Materia Medica.

L. H. Jones, M.D., Professor of Chemistry.

W. S. Elkin, M.D., Professor of Operative Surgery.

F. W. McRae, M.D, Professor of Gastro-Intestinal and Rectal Surgery.

J. Clarence Johnson, M.D., Professor of Physiology.

Virgil O. Hardon, M.D., Professor of Obstetrics and Gynecology.

J. G. Earnest, M.D., Clinical Professor of Gynecology..

F. S. Bourns, M.D., Professor of Pathology and Bacteriology.

Dunbar Roy, M.D., Clinical Professor of Diseases of Eye and Ear.

Details of the disposition of lecture rooms and dispensary department have not yet been arranged, but it is probable that the Southern building will be used for the clinics.

